# Genetic Parameters of Resilience Indicators Across Growth in Beef Heifers and Their Associations With Weight, Reproduction, Calf Performance and Pre‐Weaning Survival

**DOI:** 10.1111/jbg.70001

**Published:** 2025-06-23

**Authors:** Gustavo R. D. Rodrigues, Júlia P. S. Valente, Vanessa T. Rezende, Luis F. C. Araújo, João B. Silva Neto, Lúcio F. M. Mota, Mário L. Santana, Roberta C. Canesin, Sarah F. M. Bonilha, Lucia G. Albuquerque, Maria E. Z. Mercadante, Joslaine N. S. G. Cyrillo

**Affiliations:** ^1^ School of Agriculture and Veterinary Science São Paulo State University Jaboticabal São Paulo Brazil; ^2^ Beef Cattle Research Center Institute of Animal Science Sertãozinho São Paulo Brazil; ^3^ School of Veterinary Medicine and Animal Science University of São Paulo Pirassununga São Paulo Brazil; ^4^ Federal University of Rondonópolis Rondonópolis Mato Grosso Brazil; ^5^ National Council for Science and Technological Development Brasilia Distrito Federal Brazil

**Keywords:** adaptability, disturbances, environmental challenges, heifer pregnancy, Nellore

## Abstract

In tropical extensive beef cattle systems, heifers raised on pasture are exposed to various environmental challenges that affect their growth and reproductive performance during the first breeding. Resilience indicators derived from deviations in longitudinal traits can quantify the magnitude of these challenges and the ability of an animal to recover after disturbances. Hence, this study aimed to estimate genetic parameters for resilience indicators derived from weight deviations across growth in Nellore heifers, and their genetic correlations with yearling weight (YW), reproductive traits, calf performance and pre‐weaning survival (PWS^c^). Phenotypic records were available for 3072 heifers, while 3226 animals were genotyped with 383,856 SNP markers (after quality control). A total of 30,720 weight records were used for growth curve modelling across three developmental phases: yearling, first breeding and first calf weaning. The resilience indicators derived and analysed were as follows: (i) natural logarithm of residual variance (LnVar); (ii) lag^−1^ autocorrelation of residuals (*r*_auto_); and (iii) skewness of residuals. The weight, reproductive, calf performance and survival traits analysed were as follows: YW, weight at the beginning of the breeding season (WBS), heifer pregnancy (HP), calves birth weight (BW^c^), calves weaning weight (WW^c^), calves average daily gain from birth to weaning (ADG^c^) and PWS^c^. Genetic parameters were estimated using the ssGBLUP method under a Bayesian framework. Heritability estimates (*h* ^2^) were highest for LnVar, ranging from 0.32 ± 0.03 (calf weaning) to 0.42 ± 0.03 (breeding). Moderate *h* ^2^ values were observed for *r*_auto_ (0.22 ± 0.03 to 0.29 ± 0.03), whereas skewness had low heritability (0.08 ± 0.02 to 0.13 ± 0.02). Genetic correlations (*r*_g_) between LnVar and weight traits were unfavourable. In contrast, *r*_auto_ exhibited favourable correlations with YW (−0.29 ± 0.08 to −0.50 ± 0.08). LnVar at breeding showed favourable and moderate *r*_g_ with HP (−0.37 ± 0.10). All resilience indicators were favourably correlated with PWS^c^, with the strongest estimate observed for LnVar at calf weaning (−0.28 ± 0.15). These findings provide novel insights into the genetic basis of resilience in growing beef heifers. LnVar and *r*_auto_, in particular, emerge as promising traits for selecting animals better adapted to environmental variability. Additionally, favourable genetic correlations with fertility and survival traits suggest that more resilient heifers are more likely to become pregnant during their first breeding season and raise calves with higher survival rates until weaning.

## 1 Introduction

In extensive livestock production systems, where pasture is the primary source of nutrition, herd productivity is highly susceptible to seasonal fluctuations in forage availability. This issue becomes particularly critical during the dry season in tropical regions due to declines in pasture quality and quantity, resulting in reduced weight gain and overall performance (Montes et al. 2021; McAllister et al. 2020). Such environmental variability has a direct impact on the growth and reproductive development of heifers, which must attain an appropriate physiological and nutritional status before the beginning of the breeding season to ensure optimal fertility and reproductive success (Winton et al. 2024; Davis et al. 2023; Gwazdauskas 1985). Given that reproductive efficiency is a key determinant of the sustainability of beef cattle production, the appropriate development of heifers is of paramount importance. Consequently, the identification and selection of animals with greater resilience to environmental stressors, such as forage scarcity and heat stress, emerge as a promising approach to enhance the adaptability, efficiency and long‐term productivity of herds in pasture‐based systems (Rodrigues, Rezende, et al. 2024; Berghof et al. 2019).

Resilient animals are defined as those capable of maintaining their level of productivity under adverse conditions and/or exhibiting rapid recovery following environmental disturbances (Poppe et al. 2020; Berghof et al. 2019). The resilience capacity of an animal can be estimated by evaluating temporal deviations in performance, typically by comparing observed phenotypes with those predicted under hypothetical, stress‐free conditions using statistical modelling approaches, as proposed by Berghof et al. (2019). These deviations offer valuable insights into an individual's capacity to adapt to and recover from both anticipated and unforeseen environmental challenges, as they reflect patterns of variability over time (Colditz 2022; Berghof et al. 2019). Among the most commonly used indicators of resilience are residual variance, first‐order autocorrelation and the skewness of deviations. These metrics have been applied across various livestock species and contexts, including assessments of feed intake, weight gain and feeding behaviour in growing and finishing pigs (Gorssen et al. 2023, 2024); fluctuations in daily milk yield in lactating dairy cows (Chen et al. 2023; Poppe et al. 2020, 2021; Elgersma et al. 2018) and deviations in body weight during the growth phase of beef cattle (Rodrigues, Rezende, et al. 2024). Moreover, several studies have reported significant genetic correlations between resilience indicators and key traits associated with longevity, health and fertility in dairy cattle (Chen et al. 2023; Poppe et al. 2020).

In a previous study, Rodrigues, Rezende, et al. (2024) evaluated different statistical models to estimate growth curves and derive resilience indicators based on body weight deviations in growing beef heifers from the Nellore ( *Bos taurus indicus* ), Caracu ( *Bos taurus taurus* ) and Guzerat ( *Bos taurus indicus* ) breeds. Our findings indicated that heifers with higher yearling weight tended to exhibit increased sensitivity to environmental stressors. However, these animals also demonstrated a greater capacity for recovery and superior weight gain under favourable conditions. These results emphasise the need to consider both environmental sensitivity and recovery potential when assessing resilience, particularly in the context of genetic improvement programmes targeting beef cattle raised under variable environmental conditions.

Nevertheless, to the best of our knowledge, no studies have specifically explored the relationship between resilience capacity during growth and reproductive traits in Zebu cattle. Thus, we hypothesise that heifers which are more resilient to environmental challenges during growth will exhibit better reproductive rates than less resilient heifers. Therefore, the objective of this study was to estimate genetic parameters for resilience indicators derived from deviations in body weight across growth and to evaluate their genetic correlations with yearling weight, reproductive traits, calf performance and pre‐weaning calf survival in Nellore heifers.

## 2 Materials and Methods

### 2.1 Ethical Notes

Data were obtained from an existing database and approval of the Ethics Committee was therefore not necessary.

### 2.2 Population

All datasets used in this study were obtained from a Nellore cattle population developed and maintained within an experimental breeding programme at the Beef Cattle Research Center (Institute of Animal Science—IZ, Sertãozinho, SP, Brazil). This population consists of three distinct selection lines, each subjected to over four decades of selection, initiated in 1980, with the primary goal of selecting animals based on yearling weight (YW), measured at 378 days of age for young bulls and 550 days of age for heifers. The three established selection lines are as follows: Line 1—Nellore Control (NeC), in which animals have been selected to maintain yearling weight (YW) close to the mean of their contemporary group (CG), defined as animals born in the same year and selection line (stabilising selection); Line 2—Nellore Selection (NeS), where animals are selected for superior YW within their respective CG; and Line 3—Nellore Traditional (NeT), which was initially selected solely for higher YW but, since 2008, has adopted a multi‐trait selection index in which YW and residual feed intake (RFI) contribute equally (50% each) to selection decisions. Thus, YW has remained a core selection trait across all lines, either as a single‐trait target (NeC and NeS) or as part of a balanced breeding goal (NeT).

These selection lines are closed, meaning that only bulls and dams born within each respective line are used for breeding, except for NeT, which occasionally incorporates animals from NeS. Inbreeding levels are rigorously managed through strategic matings based on co‐ancestry coefficients (Bem et al. 2024). Despite being closed selection lines under selection for 44 years, these selection lines remain pivotal to the ongoing genetic improvement of the Nellore breed. More details about the management practices applied throughout the years (see Benfica et al. 2024; Rodrigues, Rezende, et al. 2024), effective population size and number of equivalent generations (see Rodrigues et al. 2025) and the history of the experimental breeding programme (see Cyrillo et al. 2001; Mercadante et al. 2003) can be found in previous studies from our group.

### 2.3 Phenotypic Data

A total of 3072 Nellore heifers born between 1981 and 2020, and 2157 calves born from these heifers were included in the study. The traits analysed were as follows: (i) YW; (ii) weight at the beginning of the breeding season (WBS); (iii) natural logarithm of the residual variance (LnVar) measured at yearling, breeding and calf weaning (defined in topic 2.5 and Table 2); (iv) lag^−1^ autocorrelation of residuals (*r*_auto_) measured at yearling, breeding and calf weaning (defined in topic 2.6 and Table 2); (v) skewness of residuals measured at yearling, breeding and calf weaning (defined in topic 2.5 and Table 2); (vi) heifer pregnancy (HP); (vii) birth weight from the heifer's first calf (BW^C^); (viii) weaning weight of the heifer's first calf (WW^C^); (ix) average daily gain from birth until weaning of the heifer's first calf (ADG^C^); and (x) pre‐weaning survival of the heifer's first calf (PWS^C^). Descriptive statistics for all traits are presented in Table 3.

Calves were weaned at an average age of 7 months, when they were weighed, and the WW^C^ was adjusted for 210 days of age using the following equation:

$${\mathrm{WW}}^{C}={\mathrm{BW}}^{C}+\left({\mathrm{ADG}}^{C}*210\right)$$

The heifers were managed under pasture‐based conditions, predominantly grazing on *Urochloa brizantha* cv. Marandu. Nutritional supplementation was seasonally adjusted based on forage availability. During the dry season, animals received a protein–mineral supplement containing: Crude Protein = 300 g/kg; NPN = Protein Equivalent = 226 g/kg; Calcium = 80 g/kg; Cobalt = 20 mg/kg; Copper = 250 mg/kg; Sulphur = 8000 mg/kg; Phosphorus = 20 g/kg; Iodine = 20 mg/kg; Magnesium = 4000 mg/kg; Manganese = 175 mg/kg; Selenium = 4.5 mg/kg; Sodium = 65 g/kg; Zinc = 900 mg/kg; Monensin Sodium = 300 mg/kg. During the rainy season, mineral salt supplementation was provided, offering: Calcium = 170 g/kg; Cobalt = 80 mg/kg; Copper = 1000 mg/kg; Sulphur = 22 g/kg; Phosphorus = 80 g/kg; Iodine = 80 mg/kg; Magnesium = 8 g/kg; Manganese = 700 mg/kg; Selenium = 18 mg/kg; Sodium = 120 g/kg; Zinc = 3600 mg/kg. At an average age of 550 days, the heifers were weighed, and YW was adjusted using the following equation:

$$\mathrm{YW}={\mathrm{WW}}^{C}+\left({\mathrm{ADG}}_{\mathrm{wy}}*340\right)$$

where ADG_wy_ is the average daily gain from weaning to yearling (kg/day).

The HP trait was defined by assigning the value 1 (success) to heifers that became pregnant in their first breeding season at 2 years of age and assigning 0 (failure) otherwise. The PWS of the calves was determined by assessing whether the calf survived (1) or did not survive (0) until the weaning date that occurred at approximately 7 months.

For heifer traits, CGs were formed by the combination of the birth year and selection line, resulting in 78 distinct groups. For calf traits, CGs incorporated the calf's birth year, selection line and sex, totalling 156 CGs. For continuous traits, observations beyond ±3.5 standard deviations from the CG mean were excluded. In the case of binary traits, CGs lacking within‐group variability (i.e., all animals presenting the same score) were also excluded. All CGs retained for further analyses contained at least five individuals.

To model heifers' growth curves, the dataset included 23,756 body weight records for birth‐to‐yearling, 26,812 body weight records for birth‐to‐first breeding, and 32,058 body weight records for birth‐to‐first‐calf weaning. These data were used to model growth curves corresponding to: (1) from birth to yearling (565 ± 50 days of age); (2) from birth to the first breeding season (742 ± 68 days of age); and (3) from birth to weaning of the first calf (1265 ± 98 days of age). The quality control of the weight records was based on the following criteria: (1) weights were grouped in 11 age classes based on the age of the animal at the time of weighing (as described in Table 1), and records with a difference greater than ±3.5 standard deviations from the mean of the CG were excluded; (2) heifers with less than four weight records over the study period were removed; and (3) CG with less than five individuals within each weighing class were also removed. After quality control, the dataset remained with 21,476 weight records for Phase 1, 24,548 for Phase 2 and 30,720 for Phase 3.

**TABLE 1:** Descriptive statistics of heifer weight by age class in Nellore cattle.

Age classes	Age intervals (days)	*N*	Mean ± SD
1	Birth weight	3072	30 ± 5
2	1–140	3179	121 ± 23
3	141–280	3821	172 ± 30
4	281–420	3134	203 ± 36
5	421–560	3192	266 ± 49
6	561–700	2956	308 ± 47
7	701–840	3271	341 ± 57
8	841–980	2474	410 ± 51
9	981–1120	2051	325 ± 33
10	1121–1260	1833	445 ± 62
11	1261–1400	1737	454 ± 65

Abbreviation: SD, standard deviation.

### 2.4 Genotypic Data

A total of 3226 genotyped individuals were included in this study, comprising 1485 heifers with phenotypic records and their relatives. Genotyping was performed using panels of varying densities over the years (Table S1). Individuals genotyped with medium‐density panels were imputed to a high‐density (HD) panel comprising 770 K single nucleotide polymorphism (SNP) markers. Prior to imputation, quality control was applied to all SNP panels, resulting in the exclusion of non‐autosomal markers, SNPs with a GenCall score below 0.80, monomorphic variants and those mapped to the same genomic position. The imputation process utilised a reference population of 6862 animals genotyped with the HD panel and was conducted using FImpute v3 (Sargolzaei et al. 2014). The ARS‐UCD1.2 *Bos taurus* genome assembly (Rosen et al. 2020) served as the reference map for imputation.

Following imputation, an additional quality control step was performed, which included: (1) removal of SNPs with a minor allele frequency (MAF) ≤ 0.05; (2) exclusion of SNPs and samples with a call rate ≤ 0.90; (3) elimination of SNPs deviating from Hardy–Weinberg equilibrium (HWE) with a *p* value ≤ 10^−5^; and (4) exclusion of SNPs or samples exhibiting Mendelian conflicts. After genotypes quality control, 3226 animals and 383,856 SNP markers remained in the genomic database.

### 2.5 Growth Curves Modelling

In this study, seven statistical models were used to predict growth curves across the three evaluated phases (as defined in Section 2.3). The tested models were: (i) repeated measures (linear model), (ii) Brody (nonlinear model) and (iii) models incorporating Legendre orthogonal polynomials of orders ranging from second to sixth. The fixed effects incorporated in the analyses were CG and month of birth (September–November) of the heifers, with age (in days) included as a covariate. Animals were considered a random effect, and multiple covariance matrix structures were assessed to model residual variance. Among these, the first‐order autoregressive covariance structure provided the best fit, as determined by the Akaike information criterion (AIC) and Bayesian information criterion (BIC). Each model was fitted for every animal in the dataset, and growth curves were estimated separately for each developmental phase. Consequently, these models were applied three times to estimate growth up to yearling, the beginning of the breeding season and weaning of the first calf. The optimal model for each phase was selected based on AIC and BIC criteria. All statistical analyses were conducted using RStudio version 4.3.2 (R Core Team 2022). Further details regarding the modelling of growth curves and fitting procedures can be found in Rodrigues, Rezende, et al. (2024), a previous study from our research group that evaluated the suitability of these statistical models in deriving resilience indicators for growing heifers.

### 2.6 Resilience Indicators

After estimating the weights of the animals using the growth models, weight deviations were obtained using the equation W_dev_ = W_obs_–W_est_, where W_dev_ represents the weight deviation, W_obs_ is the observed weight, and W_est_ is the estimated weight. The weight deviations were used to calculate three resilience indicators: (1) natural logarithm of the residual variance (LnVar), (2) lag^−1^ autocorrelation (*r*_auto_) and (3) skewness of weight deviations. Each of these indicators was calculated considering the weight deviations obtained from the growth models at each of the three evaluated phases (yearling, beginning of the breeding season and weaning of the first calf). Table 2 provides the definition of each resilience indicator evaluated in this study.

**TABLE 2:** Definition of the resilience indicators evaluated in this study.

Indicator	Definition
LnVar	Natural logarithm of residual variance. This indicator was calculated based on the variance of weight deviations obtained from growth models, considering specific periods: from birth to yearling (LnVar_yearling_); from birth to the first breeding season (LnVar_breeding_); and from birth to the weaning of the first calf (LnVar_calf_weaning_). A lower LnVar indicates greater resilience, suggesting that animals are less impacted by environmental challenges Berghof et al. (2019).
*r*_auto_	Lag^−1^ autocorrelation of residuals. This indicator was calculated based on the first‐order autocorrelation of weight deviations obtained from growth models, considering specific periods: from birth to yearling (*r*_auto_yearling_); from birth to the first breeding season (*r*_auto_breeding_); and from birth to the weaning of the first calf (*r*_auto_calf_weaning_). A *r*_auto_ close to 0 indicates greater resilience—animals with a higher recovery rate Berghof et al. (2019).
Skewness	Skewness of residuals (weight deviations). This indicator was calculated considering the specific phases: from birth to yearling (skewness_yearling_); from birth to the first breeding season (skewness_breeding_); and from birth to the weaning of the first calf (skewness_calf_weaning_). A skewness close to 0 indicates greater resilience—balance between performance losses and gains Berghof et al. (2019).

**TABLE 3:** Descriptive statistics of the traits evaluated.

Traits	*N*	Mean	SD	Min	Max
YW (kg)	3072	295.51	44.29	159.87	434.44
WBS (kg)	3072	333.40	52.55	168.00	514.00
HP (%)^a^	3072	72.08	—	0.00	100.00
LnVar_yearling_	3072	5.78	0.92	3.25	8.20
LnVar_breeding_	3072	6.18	0.85	3.13	8.52
LnVar_calf_weaning_	1964	6.70	0.64	4.69	8.47
*r*_auto_yearling_	3072	0.19	0.28	−0.63	0.70
*r*_autobreeding_	3072	0.17	0.30	−0.70	0.73
*r*_auto_calf_weaning_	1964	0.10	0.29	−0.69	0.78
Skewness_yearling_	3072	0.09	0.58	−1.46	1.65
Skewness_breeding_	3072	0.05	0.69	−1.64	1.81
Skewness_calf_weaning_	1964	−0.13	0.65	−1.73	1.65
BW^C^ (kg)	2157	29.71	4.66	16.00	43.00
WW^C^ (kg)	1964	179.92	29.30	100.11	261.70
ADG^C^ (kg/day)	1964	0.72	0.13	0.35	1.08
PWS^C^ (%)^a^	2157	89.05	—	0.00	100.00

Abbreviations: ADG^C^, average daily gain from birth to weaning of the heifer's calf; BW^C^, birth weight of the heifer's calf; HP, heifer pregnancy; LnVar, natural logarithm of residual variance; PWS^C^, pre‐weaning survival of the heifer's calf; *r*_auto_, lag^−1^ autocorrelation of residuals; skewness, skewness of residuals; WBS, weight at the beginning of the breeding season; WW^C^, weaning weight; YW, yearling weight.

^a^ For HP and PWS, the mean represents the frequency of event occurrences relative to the number of observations.

**TABLE 4:** Variance components and heritability (*h* ^2^) estimates (± standard deviation) of the traits evaluated.

Trait	$\sigma_{a}^{2}$	$\sigma_{m}^{2}$	$\sigma_{e}^{2}$	*h* ^2^
YW	315.85 ± 28.22	—	320.21 ± 17.58	0.50 ± 0.03
WBS	350.24 ± 29.62	—	360.32 ± 18.53	0.49 ± 0.03
HP	0.078 ± 0.040	—	0.984 ± 0.034	0.07 ± 0.04
LnVar_yearling_	0.257 ± 0.025	—	0.448 ± 0.018	0.37 ± 0.03
LnVar_breeding_	0.252 ± 0.023	—	0.342 ± 0.015	0.42 ± 0.03
LnVar_calf_weaning_	0.125 ± 0.013	—	0.268 ± 0.010	0.32 ± 0.03
*r*_auto_yearling_	0.012 ± 0.002	—	0.042 ± 0.002	0.22 ± 0.03
*r*_autobreeding_	0.017 ± 0.002	—	0.043 ± 0.003	0.29 ± 0.03
*r*_auto_calf_weaning_	0.014 ± 0.002	—	0.056 ± 0.002	0.20 ± 0.02
Skewness_yearling_	0.028 ± 0.007	—	0.266 ± 0.007	0.10 ± 0.02
Skewness_breeding_	0.040 ± 0.007	—	0.261 ± 0.008	0.13 ± 0.02
Skewness_calf_weaning_	0.057 ± 0.009	—	0.632 ± 0.08	0.08 ± 0.02
BW^C^	6.43 ± 1.40	2.26 ± 0.82	7.53 ± 0.89	0.39 ± 0.05
WW^C^	92.22 ± 35.64	17.05 ± 14.01	360.98 ± 30.25	0.17 ± 0.06
ADG^C^	0.002 ± 0.001	0.002 ± 0.001	0.009 ± 0.001	0.16 ± 0.06
PWS^C^	0.352 ± 0.265	0.358 ± 0.245	0.978 ± 0.045	0.15 ± 0.06

Abbreviations: $\sigma_{a}^{2}$, direct additive genetic variance; $\sigma_{e}^{2}$, residual variance; $\sigma_{m}^{2}$, maternal additive genetic variance; ADG^C^, average daily gain from birth to weaning of the heifer's calf; BW^C^, birth weight of the heifer's calf; HP, heifer pregnancy; LnVar, natural logarithm of residual variance; PWS^C^, pre‐weaning survival of the heifer's calf; *r*_auto_, lag^−1^ autocorrelation of residuals; skewness, skewness of residuals; WBS, weight at the beginning of the breeding season; WW^C^, weaning weight of the heifer's calf; YW, yearling weight.

**TABLE 5:** Genetic correlations (above the diagonal) and phenotypic correlations (below the diagonal) between the resilience indicators (estimates ± standard deviation).

Trait	LnVar_yearling_	LnVar_breeding_	LnVar_calf_weaning_	*r*_auto_yearling_	*r*_auto_breeding_	*r*_auto_calf_weaning_	Skewness_yearling_	Skewness_breeding_	Skewness_calf_weaning_
LnVar_yearling_		0.95 ± 0.01	0.90 ± 0.02	−0.69 ± 0.06	−0.50 ± 0.09	−0.43 ± 0.10	0.47 ± 0.10	0.31 ± 0.10	0.25 ± 0.09
LnVar_breeding_	0.85 ± 0.01		0.97 ± 0.01	−0.53 ± 0.08	−0.74 ± 0.06	−0.50 ± 0.11	0.36 ± 0.10	0.48 ± 0.10	0.22 ± 0.09
LnVar_calf_weaning_	0.66 ± 0.01	0.82 ± 0.01		−0.62 ± 0.06	−0.65 ± 0.06	−0.72 ± 0.06	0.34 ± 0.11	0.40 ± 0.10	0.45 ± 0.10
*r*_auto_yearling_	−0.56 ± 0.01	−0.43 ± 0.01	−0.23 ± 0.01		0.90 ± 0.02	0.73 ± 0.06	−0.14 ± 0.08	−0.10 ± 0.09	0.11 ± 0.09
*r*_auto_breeding_	−0.30 ± 0.01	−0.50 ± 0.01	−0.24 ± 0.01	0.80 ± 0.01		0.95 ± 0.01	−0.11 ± 0.12	−0.13 ± 0.11	−0.05 ± 0.10
*r*_auto_calf_weaning_	−0.13 ± 0.01	−0.23 ± 0.01	−0.30 ± 0.01	0.56 ± 0.01	0.75 ± 0.01		−0.08 ± 0.14	−0.14 ± 0.12	−0.20 ± 0.10
Skewness_yearling_	0.14 ± 0.02	0.11 ± 0.02	0.10 ± 0.02	−0.08 ± 0.02	−0.02 ± 0.01	0.01 ± 0.01		0.84 ± 0.08	0.65 ± 0.10
Skewness_breeding_	0.13 ± 0.02	0.14 ± 0.02	0.13 ± 0.02	−0.05 ± 0.02	−0.04 ± 0.01	0.03 ± 0.01	0.64 ± 0.01		0.92 ± 0.03
Skewness_calf_weaning_	0.10 ± 0.02	0.10 ± 0.02	0.18 ± 0.01	−0.09 ± 0.02	−0.03 ± 0.01	0.09 ± 0.01	0.34 ± 0.01	0.68 ± 0.01	

Abbreviations: LnVar, natural logarithm of residual variance; *r*_auto_ lag^−1^ autocorrelation of residuals; skewness, skewness of residuals.

**TABLE 6:** Genetic correlations between the resilience indicators with weights, reproduction traits, calf performance and pre‐weaning survival (estimates ± standard deviation).

Traits	YW	WBS	HP	BW^C^	WW^C^	ADG^C^	PWS^C^
LnVar_yearling_	0.70 ± 0.05	0.61 ± 0.05	−0.27 ± 0.10	0.35 ± 0.10	0.49 ± 0.15	0.45 ± 0.15	−0.06 ± 0.15
LnVar_breeding_	0.60 ± 0.05	0.64 ± 0.05	−0.37 ± 0.10	0.47 ± 0.10	0.63 ± 0.15	0.44 ± 0.15	−0.17 ± 0.15
LnVar_calf_weaning_	0.57 ± 0.06	0.58 ± 0.06	−0.36 ± 0.10	0.47 ± 0.09	0.68 ± 0.15	0.45 ± 0.14	−0.28 ± 0.15
*r*_auto_yearling_	−0.40 ± 0.08	−0.34 ± 0.08	−0.12 ± 0.12	−0.17 ± 0.11	0.08 ± 0.18	−0.04 ± 0.18	−0.21 ± 0.14
*r*_auto_breeding_	−0.32 ± 0.07	−0.36 ± 0.07	−0.16 ± 0.11	−0.33 ± 0.11	−0.14 ± 0.16	−0.07 ± 0.15	−0.22 ± 0.15
*r*_auto_calf_weaning_	−0.29 ± 0.08	−0.33 ± 0.08	−0.03 ± 0.12	−0.19 ± 0.10	−0.03 ± 0.17	−0.06 ± 0.15	−0.23 ± 0.15
Skewness_yearling_	0.30 ± 0.10	0.21 ± 0.09	−0.15 ± 0.10	0.43 ± 0.10	0.55 ± 0.15	0.21 ± 0.14	−0.05 ± 0.15
Skewness_breeding_	0.24 ± 0.10	0.29 ± 0.09	−0.16 ± 0.09	0.41 ± 0.11	0.60 ± 0.15	0.37 ± 0.15	−0.15 ± 0.15
Skewness_calf_weaning_	0.15 ± 0.09	0.17 ± 0.08	−0.10 ± 0.11	0.46 ± 0.12	0.65 ± 0.15	0.38 ± 0.15	−0.16 ± 0.15

Abbreviations: ADG^C^, average daily gain from birth to weaning of the heifer's calf; BW^C^, birth weight of the heifers's calf; HP, heifer pregnancy; LnVar, natural logarithm of residual variance; PWS^C^, pre‐weaning survival of the heifer's calf; *r*_auto_, lag^−1^ autocorrelation of residuals; skewness, skewness of residuals; WBS, weight at the beginning of the breeding season; WW^C^, weaning weight of the heifer's calf; YW, yearling weight.

### 2.7 Genetic Analyses

The genetic analyses were performed considering a Bayesian framework and the single‐step genomic best linear unbiased prediction (ssGBLUP) method, which consider the pedigree‐genomic relationship matrix (H) and its inverse (H^−1^) is given as described by Aguilar et al. (2010):

$$H^{-1}=A^{-1}+\left[\begin{array}{cc} 0 & 0\\ 0 & G^{-1}-A_{22}^{-1} \end{array}\right]$$

where $A^{-1}$ is the inverse of the pedigree‐based relationship matrix for all animals and $A_{22}^{-1}$ is the inverse for the genotyped animals; and $G^{-1}$ is the inverse of the genomic relationship matrix (G) described by VanRaden (2008):

$$G=\frac{{\mathrm{MM}}^{\prime }}{2\sum p_{i}\left(1-p_{i}\right)}$$

where **M** is the matrix of SNP markers with codes 0, 1 and 2 for the genotypes AA, AB and BB, respectively, adjusted to the allelic frequencies expressed as 2*pi*; and **p**_**i**_ is the frequency of the ith SNP marker.

All traits were evaluated using a single‐trait animal model to estimate heritability and a bivariate animal model to estimate genetic correlations. To integrate the genetic evaluation model, all systematic effects and covariates were tested for significance in relation to the traits studied using the *lm* (linear traits) and glm (binary traits) functions in R software (R Core Team 2022). Effects that exhibited a significant effect on the traits (*p* value < 0.05), were considered in the model. For traits measured in heifers, the systematic effects considered were CGs, month of birth (9–11) and cow age (covariate with linear and quadratic effects). For traits measured in calves, the systematic effects included CG, month of birth of the calves (September, October and November) and the heifer's age at calving (covariate with linear and quadratic effects). The random effects included direct additive genetic, maternal additive genetic (only for traits measured in calves) and residual effects.

The univariate model for traits measured in heifers can be expressed in matrix notation as follows:

$$y=\mathrm{X\beta}+Z_{1}a+e$$

where y is the vector of observations for each trait; β is the vector with solutions for the systematic effects and covariates; a is the vector with the solutions for the direct additive genetic effect, assumed as a ~ *N*(0, $H\sigma_{a}^{2}$), where **H** is the relationship matrix that combines pedigree (A) and genomic (G) information, and $\sigma_{a}^{2}$ is the genetic additive variance; e is the vector of residuals effects, assumed as e ~ *N*(0, $I\sigma_{e}^{2}$), where **I** is an identity matrix and $\sigma_{e}^{2}$ is the residual variance; **X** is the incidence matrix that relates β and y; and **Z**_**1**_ is the incidence matrix that relates **a** and **y**.

The univariate model for traits measured in calves can be expressed in matrix notation as follows:

$$y=\mathrm{X\beta}+Z_{1}a+Z_{2}m+e$$

where m is the vector with the solutions for the maternal additive genetic effect, assumed as m ~ *N*(0, $H\sigma_{m}^{2}$), and Z_2_ is the incidence matrix that relates m and y. The direct additive genetic and maternal additive genetic effects were considered to be correlated based on Cov(**a**, **m**) = **Hσ**_**am**_, where **σ**_**am**_ is the covariance between **a** and **m**. We did not include the maternal permanent environmental effect because this study only considered calves born to heifers, who did not have repeated calving records.

For binary traits (HP and PWS), the univariate model was fitted via a threshold model, assuming an underlying distribution was used as follows:

$$f\left(y|l_{i}\right)=\prod_{i=1}^{n_{i}}1\left(l_{i}<t_{i}\right)1\left(y=0\right)+1\left(l_{i}>t_{i}\right)1\left(y=1\right)$$

where y is the binary trait, $l_{i}$ is the underlying liability of observation i; $t_{i}$ is the threshold that defines the category response for the binary traits, and n_i_ corresponds to the number of observations.

To estimate the genetic correlations between traits, a bivariate animal model was used as follows:

$$\left[\begin{array}{c} y_{1}\\ y_{2} \end{array}\right]=\left[\begin{array}{cc} X_{1} & 0\\ 0 & X_{2} \end{array}\right]\left[\begin{array}{c} \beta_{1}\\ \beta_{2} \end{array}\right]+\left[\begin{array}{cc} Z_{11} & 0\\ 0 & Z_{12} \end{array}\right]\left[\begin{array}{c} a_{1}\\ a_{2} \end{array}\right]+\left[\begin{array}{cc} Z_{21} & 0\\ 0 & Z_{22} \end{array}\right]\left[\begin{array}{c} m_{1}\\ m_{2} \end{array}\right]\left[\begin{array}{c} e_{1}\\ e_{2} \end{array}\right]$$

where **y**_**1**_ and **y**_**2**_ represent vectors with phenotypes for the Traits 1 and 2, respectively; $\beta_{1}$ and $\beta_{2}$ are vectors with the solutions for the systematic effects and covariates; **a**_**1**_ and **a**_**2**_ are vectors with solutions for the direct additive genetic effects, assumed as: $\left[\begin{array}{c} a_{1}\\ a_{2} \end{array}\right]\sim N\left(\left[\begin{array}{c} 0\\ 0 \end{array}\right],\;H⊗\left[\begin{array}{cc} \sigma_{a1}^{2} & \sigma_{a1a2}\\ \sigma_{a1a2} & \sigma_{a2}^{2} \end{array}\right]\right)$, where $\sigma_{a1}^{2}$ is the direct additive genetic variance of Trait 1, $\sigma_{a2}^{2}$ is the direct additive genetic variance of Trait 2, $\sigma_{a1a2}$ is the direct additive genetic covariance between Traits 1 and 2, and ⊗ is the kronecker product; m_1_ and m_2_ are vectors with the solutions for the maternal additive genetic effects, considered only for traits measured in calves, assumed as: $\left[\begin{array}{c} m_{1}\\ m_{2} \end{array}\right]\sim N\left(\left[\begin{array}{c} 0\\ 0 \end{array}\right],\;H⊗\left[\begin{array}{cc} \sigma_{m1}^{2} & \sigma_{m1m2}\\ \sigma_{m1m2} & \sigma_{m2}^{2} \end{array}\right]\right)$, where $\sigma_{m1}^{2}$ is the maternal additive genetic variance of Trait 1, $\sigma_{m2}^{2}$ is the maternal additive genetic variance of Trait 2, $\sigma_{m1m2}$ is the maternal additive genetic covariance between Traits 1 and 2, **e**_**1**_ and **e**_**2**_ are vectors of residual effects, assumed as $\left[\begin{array}{c} e_{1}\\ e_{2} \end{array}\right]\sim N\left(\left[\begin{array}{c} 0\\ 0 \end{array}\right],\;I⊗\left[\begin{array}{cc} \sigma_{e1}^{2} & \sigma_{e1e2}\\ \sigma_{e1e2} & \sigma_{e2}^{2} \end{array}\right]\right)$, where **I** is the identity matrix, $\sigma_{e1}^{2}$ is the residual variance of Trait 1, $\sigma_{e2}^{2}$ is the residual variance of Trait 2, and $\sigma_{e1e2}$ is the residual covariance between Traits 1 and 2; **X**_**1**_ and **X**_**2**_ are incidence matrices tor the systematic effects for Traits 1 and 2, respectively; **Z**_**11**_ and **Z**_**12**_ are incidence matrices for the direct additive genetic effects for Traits 1 and 2, respectively; and **Z**_**21**_ and **Z**_**22**_ are incidence matrices for the maternal additive genetic effects for calves Traits 1 and 2, respectively.

Genetic analyses were conducted using the gibbsf90+ and postgibbsf90 software (Misztal et al. 2022) to obtain the posterior distributions of genetic parameters. The Gibbs sampling consisted of a chain of 1,500,000 cycles, with a burn‐in of the first 150,000 iterations and storage every 50 cycles, totalling 27,300 samples for the estimation of (co)variance components and genetic parameters. The convergence of the estimates obtained by the Monte Carlo Markov Chain method was checked using the Geweke test (Geweke 1991) and visual inspection through the BOA package available in R (Smith 2007).

## 3 Results and Discussion

### 3.1 Modelling Heifer Resilience Across Growth Phases Using Longitudinal Weight Data

The primary objective of this study was to investigate a potential association between heifers' resilience to environmental challenges during growth and their reproductive efficiency during their first breeding. There is a notable gap in beef cattle research regarding the assessment of resilience to environmental disturbances based on deviations in longitudinally measured traits. This aspect is particularly relevant given the increasing impact of climate change on livestock production (Berghof et al. 2019). A comprehensive understanding of the relationship between resilience, growth and reproduction in beef cattle is essential for establishing selection criteria that promote animals adapted to their environment while meeting specific breeding objectives.

**FIGURE 1:**
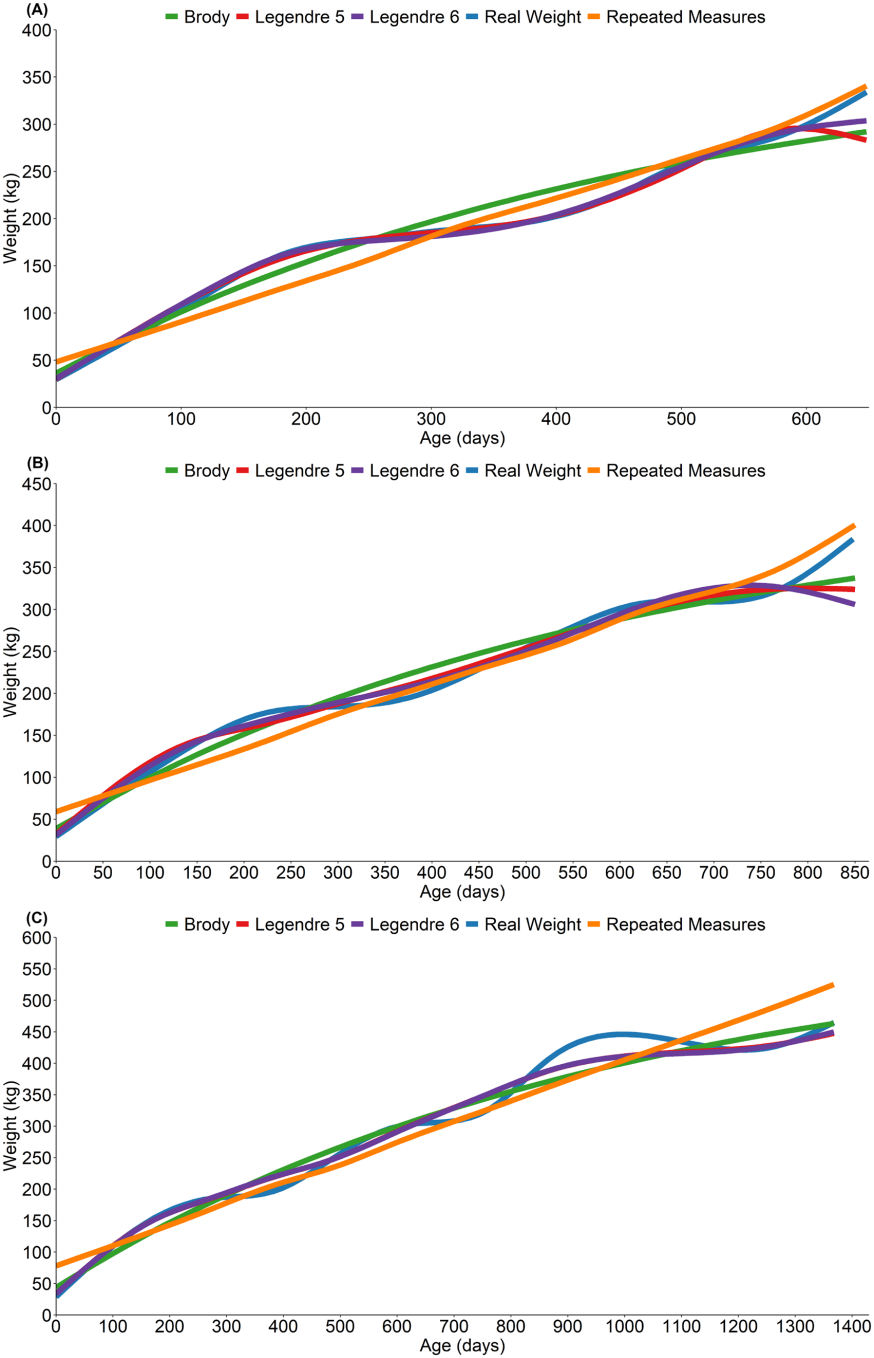
Growth curves modelling for Nellore heifers. (A) growth from birth until yearling; (B) growth from birth until the first breeding season; (C) growth from birth until the weaning of first calf. [Colour figure can be viewed at wileyonlinelibrary.com]

Among the evaluated statistical models for estimating growth curves of Nellore heifers, the sixth‐order orthogonal Legendre polynomials model (fifth‐degree regression) had the best predictive performance across all growth phases, as determined by the AIC and BIC criteria (Tables S2–S4). Figure 1 presents the estimated growth curves of the animals throughout the three evaluated phases. To maintain clarity, growth curves from models using Legendre polynomials of Orders 2 to 4 were omitted, with only weight estimates from Orders 5 and 6 presented.

### 3.2 Heritability Estimates

Table 4 displays the estimates of direct additive genetic variance ($\sigma_{a}^{2}$), maternal additive genetic variance ($\sigma_{m}^{2}$) and residual variance ($\sigma_{e}^{2}$), and heritability (*h* ^2^) for all evaluated traits. Among the resilience indicators, LnVar showed the highest *h* ^2^ estimates across all three evaluated phases, with values of 0.37 ± 0.03 (LnVar_yearling_), 0.42 ± 0.03 (LnVar_breeding_) and 0.32 ± 0.03 (LnVar_calf_weaning_). This indicator is calculated based on the variability of weight deviations throughout the growth period. Thus, animals with lower LnVar are more resilient to environmental challenges, as they can maintain growth close to that estimated by the growth models, being less impacted by disturbances. This finding indicates that this trait has enough genetic variability and may respond rapidly to genetic selection, enabling its inclusion in animal breeding programmes. Using fluctuations in daily milk production, some studies in dairy cattle reported *h* ^2^ estimates for LnVar of 0.24 ± 0.01 (Poppe et al. 2020), 0.25 ± 0.01 (Wang et al. 2022) and 0.15 ± 0.01 (Chen et al. 2023), respectively. In growing pigs, *h* ^2^ estimate of 0.23 ± 0.04 were found considering the LnVar of deviations in feed intake as a function of age (Gorssen et al. 2023). For finishing pigs, Gorssen et al. (2024) reported *h* ^2^ estimate of 0.36 ± 0.08 for LnVar of weight deviations during growth in the finishing phase. Considering beef cattle, Rodrigues, Rezende, et al. (2024) found *h* ^2^ estimates of 0.38 ± 0.04 for LnVar of weight deviations during heifer's growth until the rearing phase in Nellore cattle.

The heritability estimates for *r*_auto_ were 0.22 ± 0.03, 0.29 ± 0.03 and 0.20 ± 0.02 for *r*_auto_yearling_, *r*_auto_breeding_ and *r*_auto_calf_weaning_, respectively. The *r*_auto_ is a resilience indicator that reflects the recovery rate of animals after being exposed to environmental challenges, as its calculation is based on the autocorrelation between weight deviations over the growth period. An application of genetic selection for heifers with a higher recovery rate after experiencing a disturbance may be relevant for extensive beef cattle farming, especially considering the seasonal variation in forage production throughout the year due to tropical climatic conditions. Poppe et al. (2020) and Chen et al. (2023) reported *h* ^2^ estimates for *r*_auto_ considering fluctuations in daily milk production of 0.10 ± 0.01 and 0.06 ± 0.01, respectively.

For the skewness indicator, *h* ^2^ estimates ranged from 0.08 ± 0.02 (Skewness_calf_weaning_) to 0.13 ± 0.02 (Skewness_breeding_). Skewness is a resilience indicator proposed to reflect the effects of the environment on animal performance, where positive skewness values are associated with a positive environmental effect and negative values represent negative environmental effects (Berghof et al. 2019; Poppe et al. 2020). Other studies with dairy cattle have also found low *h* ^2^ estimates for skewness of residual daily milk production, with estimate of 0.02 ± 0.01 (Poppe et al. 2020). In pigs, a *h* ^2^ estimate of 0.03 ± 0.01 was reported for skewness of weight deviations during growth (Gorssen et al. 2023).

The YW and WBS presented high *h* ^2^ estimates, with values of 0.50 ± 0.03 and 0.49 ± 0.03, respectively. Weight traits are additively influenced by various *loci* across the bovine genome, which provides greater genetic variability and response to selection for these traits (Carvalho et al. 2020; Silva Neto et al. 2023). Some studies have already reported moderate to high *h* ^2^ estimates for weight traits measured in different ages in Nellore cattle (Portes et al. 2020; Silva Neto et al. 2020). Heifer pregnancy had a low *h* ^2^ estimate of 0.07 ± 0.04, suggesting little genetic variability and great environmental influence on the expression of this trait. Conception success in cattle is highly influenced by environmental factors (Mota et al. 2020) and body condition score during breeding (Brunes et al. 2022), as both factors play a crucial role in determining reproductive performance (D'Occhio et al. 2019; Mota et al. 2022).

Regarding calves‐related traits, BW^C^, WW^C^, ADG^C^ and PWS^C^ exhibited *h* ^2^ estimates of 0.39 ± 0.05, 0.17 ± 0.06, 0.16 ± 0.06 and 0.15 ± 0.06, respectively. Other studies with the Nellore breed have also reported low to moderate magnitude *h* ^2^ estimates for BW^C^ (0.34 ± 0.03) (Koetz et al. 2019), WW^C^ (0.21 ± 0.01) (Negreiros et al. 2024) and ADG^C^ (0.18 ± 0.03) (Koetz et al. 2019), respectively. The lower *h* ^2^ estimate for PWS^C^ indicates that a large part of the phenotypic variation in calf survival until weaning is explained by non‐genetic factors, such as birth weight, vigour, weather conditions and potential diseases (Schmidek et al. 2013). Therefore, genetic selection aimed at improving calf survival would result in low genetic gain across generations, given the low genetic variability of this trait. Some studies have reported low heritability estimates for pre‐weaning mortality in the Nellore breed, including Silva et al. (2017) (0.07 ± 0.05), Schmidek et al. (2013) (0.10 ± 0.05) and Rodrigues, Brito, et al. (2024) (0.11 ± 0.03).

### 3.3 Genetic Correlations Between the Different Resilience Indicators

The genetic correlations (*r*_g_) between LnVar measured at yearling, breeding and weaning of the first calf were 0.90 ± 0.02 (LnVar_yearling_ × LnVar_calf_weaning_), 0.95 ± 0.01 (LnVar_yearling_ × LnVar_breeding_) and 0.97 ± 0.01 (LnVar_breeding_ × LnVar_calf_weaning_), respectively (Table 5). For *r*_auto_, observed *r*_g_ values were 0.73 ± 0.06 (*r*_auto_yearling_ × *r*_auto_calf_weaning_), 0.90 ± 0.02 (*r*_auto_yearling_ × *r*_auto_breeding_) and 0.95 ± 0.01 (*r*_auto_breeding_ × *r*_auto_calf_weaning_), respectively. The skewness indicator showed *r*_g_ estimates with values ranging from 0.65 ± 0.10 (skewness_yearling_ × skewness_calf_weaning_) to 0.92 ± 0.03 (skewness_breeding_ × skewness_calf_weaning_). These results indicate that genetic selection for more resilient heifers at any of the three growth phases studied will also result in more resilient heifers at the other phases. Thus, the selection for resilient heifers can be accelerated at the yearling stage, without the need to wait until the breeding season or the weaning of the first calf to assess heifer resilience using deviations of growth curves. Chen et al. (2023) observed genetic correlations of 0.95 ± 0.06 between LnVar measured in second and third Holstein lactations, and 0.75 ± 0.19 between *r*_auto_ measured in first and third lactations.

Another finding was that genetic correlations between the same resilience indicator measured at different phases were stronger between adjacent periods, particularly when involving the breeding season, which represents an intermediate stage between yearling and the weaning of the first calf. In contrast, correlations were weaker between more distant phases, such as between yearling and the weaning of the first calf. This pattern indicates a temporal decay in genetic association as the interval between measurements increases. This may indicate that the resilience capacity of heifers at older ages, not considered in the present study, might not be highly genetically correlated with these indicators calculated during growth. However, further studies are needed to confirm this hypothesis.

Negative and unfavourable genetic correlations were observed between LnVar and *r*_auto_, with estimates ranging from −0.43 ± 0.10 (LnVar_yearling_ × *r*_auto_calf_weaning_) to −0.74 ± 0.06 (LnVar_breeding_ × *r*_auto_breeding_) (Table 5). In other words, heifers less impacted by environmental challenges (lower LnVar) also have a lower recovery rate when exposed to an environmental disturbance (higher *r*_auto_). This result highlights the importance of considering both resilience indicators in a selection index to select heifers that are less influenced by the environment, while also exhibiting rapid recovery when exposed to a disturbance. Poppe et al. (2021) using daily milk yield residuals also found unfavourable genetic correlations between LnVar and *r*_auto_, with estimates of −0.13 ± 0.02.

Regarding the genetic correlations between LnVar and skewness, positive and favourable estimates ranging from 0.22 ± 0.09 (LnVar_breeding_ × Skewness_calf_weaning_) to 0.48 ± 0.10 (LnVar_breeding_ × Skewness_breeding_) were observed (Table 4). These results highlight that genetic selection for lower LnVar also implies lower skewness, resulting in heifers that are less influenced by disturbances and that maintain a balance between performance losses or gains due to environmental challenges during growth. Other authors have reported genetic correlation estimates of 0.51 ± 0.12 between LnVar and skewness considering daily milk yield residuals (Poppe et al. 2020). Gorssen et al. (2023) found genetic correlations of 0.32 ± 0.11 between LnVar and skewness considering weight residuals in growing pigs.

Negative genetic correlations with null and weak magnitudes between *r*_auto_ and skewness were observed, with estimates ranging from −0.05 ± 0.10 (*r*_auto_breeding_ × Skewness_calf_weaning_) to −0.20 ± 0.10 (*r*_auto_calf_weaning_ × Skewness_calf_weaning_), indicating that a small portion of the genes influencing the genetic variation of *r*_auto_ are also associated with the genetic variation of skewness. Gorssen et al. (2023) found genetic correlations of −0.10 ± 0.11 between *r*_auto_ and skewness considering weight deviations during pig growth. Poppe et al. (2020) reported genetic correlation estimates of −0.20 ± 0.06 between *r*_auto_ and skewness in Holstein cattle during the lactation period.

### 3.4 Genetic Correlations Between the Resilience Indicators With Weight, Reproductive Traits, Calf Performance and Pre‐Weaning Survival

Table 6 presents the estimated *r*_g_ between the resilience indicators, YW, reproductive traits, calf performance and pre‐weaning survival. The genetic correlations between LnVar and YW ranged from 0.57 ± 0.06 (LnVar_calf_weaning_ × YW) to 0.70 ± 0.05 (LnVar_yearling_ × YW). Regarding genetic correlations between LnVar and WBS, the highest value was observed considering LnVar_breeding_ (0.64 ± 0.05). These estimates are of a high magnitude and unfavourable, indicating that heifers with higher weights are more affected by environmental disturbances (higher LnVar). However, favourable negative genetic correlations between *r*_auto_ with YW (−0.29 ± 0.08 to −0.40 ± 0.08) and WBS (−0.33 ± 0.08 to −0.36 ± 0.07) were found (Table 6), highlighting that genetic selection for higher YW results in heifers with a higher recovery rate following an environmental challenge (*r*_auto_ close to 0). These results can be explained by the compensatory gain observed in extensively raised cattle during the rainy season of the year, when forage production is abundant (Ryan et al. 1993; O'Donovan 1984). Therefore, heifers selected for higher weights may be more affected by environmental challenges but exhibit a higher recovery rate post‐challenge.

Understanding the relationship between the resilience capacity of beef heifers during growth and economically important traits is crucial for determining whether more resilient individuals can be more productive or not. Studies with Holstein cattle have shown unfavourable genetic correlations between LnVar and milk production at 305 days (0.64) (Poppe et al. 2021), as well as between *r*_auto_ and average daily milk production (0.28 ± 0.08) (Chen et al. 2023). These findings suggest that more productive individuals are generally more demanding in terms of nutritional requirements for maintenance and, consequently, are less resilient to environmental challenges (Berghof et al. 2019; Chen et al. 2023; Poppe et al. 2021). However, in beef cattle, this association between productivity and resilience has not been explored, especially using deviations of longitudinally measured traits, such as weight during growth.

Genetic correlations between skewness with YW and WBS were positive and not favourable (Table 6), ranging from weak (0.15 ± 0.09, Skewness_calf_weaning_ × YW) to moderate magnitudes (0.30 ± 0.10, Skewness_yearling_ × YW). This finding suggests that genetic selection aimed at enhancing production traits, such as yearling weight, may impact the ability of heifers to cope with environmental challenges. Poppe et al. (2020) reported genetic correlations of 0.30 ± 0.07 between skewness and average daily milk yield, suggesting that cows more resilient during lactation may be less productive. Some studies have reported that animals specialised for higher production may be more susceptible to performance loss due to heat waves (Lees et al. 2019), endoparasite infections (Gouveia et al. 2023) and subclinical diseases (Raboisson et al. 2014). This increased susceptibility may be attributed to the fact that, when exposed to a disturbance, these animals may redirect part of the energy allocated to production towards maintaining homeostasis. As a result, their performance is more affected by environmental conditions, and they require more favourable environments to achieve optimal productivity.

Genetic correlations estimated between LnVar and HP were moderate (−0.27 ± 0.10 to −0.37 ± 0.10; Table 6) and favourable. This result highlights that heifers more resilient to environmental challenges during the growth period are more likely to get pregnant during their first breeding season. This is the first finding in beef cattle that associates resilience indicators, measured by deviations in growth trajectory, with reproductive aspects of heifers. Thus, genetic selection for heifers less impacted by the environment during growth (lower LnVar) would provide a higher probability of pregnancy in these animals due to the correlated response. Null and low genetic correlations between the resilience indicators *r*_auto_ (−0.03 ± 0.12 to −0.16 ± 0.11) and skewness (−0.10 ± 0.11 to −0.16 ± 0.09) with HP were found (Table 6). The estimates reported were favourable, suggesting that even in small proportions, genetic selection for heifers with higher recovery rates after a disturbance (*r*_auto_ close to 0) and more adapted to environmental challenges (skewness close to 0) may result in animals with higher pregnancy probability in the first breeding. The genetic correlations between resilience indicators and heifer pregnancy are important for beef cattle breeding programmes in tropical regions. Given that a large portion of cattle rearing is conducted extensively, and climatic conditions are highly influential, selecting more resilient heifers could be a viable strategy to obtain animals better adapted to environmental challenges. Consequently, these animals would exhibit improved reproductive indices, as observed in this study.

Regarding the results involving genetic correlations between LnVar and the traits BW^C^, WW^C^ and ADG^C^, the highest estimates were between LnVar_calf_weaning_ × BW^C^ (0.47 ± 0.09), LnVar_calf_weaning_ × WW^C^ (0.68 ± 0.15) and LnVar_calf_weaning_ × ADG^C^ (0.45 ± 0.14), respectively (Table 6). These indicate that heifers less resilient to environmental challenges have more productive calves. One explanation could be that heavier calves consume more feed, which demands more energy from the heifer for milk production (Berry et al. 2016). This increased maintenance requirement may make these heifers more susceptible to environmental challenges, leading to greater weight variation during this growth phase. These results also support the hypothesis that animals selected for higher productivity are more susceptible to their rearing environment, as reported in other studies with dairy cattle (Poppe et al. 2020; Chen et al. 2023) and beef cattle (Rodrigues, Rezende, et al. 2024).

Favourable negative genetic correlations between *r*_auto_ and the traits BW^C^, WW^C^ and ADG^C^ were observed (Table 6). The estimates were null to low in magnitude and highlighted that heifers with a greater capacity for recovery after facing an environmental challenge may have more productive calves, with higher weights and greater pre‐weaning weight gains. After calving, the energetic balance in cattle is negative due to the mobilisation of energy for milk production for the calf, which influences both the productive aspects of the mother and the calf's performance (Berry et al. 2016). Therefore, genetic selection for more resilient animals, considering the *r*_auto_ indicator, would be beneficial for obtaining heifers that are more adapted to the environment and potentially capable of weaning heavier calves, based on the genetic correlations observed in the present study. The genetic correlations between the skewness indicator and pre‐weaning production traits of the calves were also stronger when considering Skewness_calf_weaning_, with estimates of 0.46 ± 0.12 (Skewness_calf_weaning_ × BW^C^), 0.65 ± 0.15 (Skewness_calf_weaning_ × W210^C^) and 0.38 ± 0.15 (Skewness_calf_weaning_ × ADG^C^). This association supports the hypothesis that heifers weaning heavier calves are more susceptible to environmental challenges.

Favourable genetic correlations were observed between heifer resilience indicators and pre‐weaning calf survival. The strongest estimates were observed when considering the growth phase of heifers until the weaning of their first calf, with moderate genetic correlation estimates ranging from −0.28 ± 0.15 (LnVar_calf_weaning_ × PWS^C^) to −0.16 ± 0.15 (Skewness_calf_weaning_ × PWS^C^). These findings highlight that genetic selection for more resilient heifers during growth would also result in genetic gains in traits associated with reproductive efficiency, such as higher HP success and calf survival until weaning.

Considering all the results reported in this study, it is important to emphasise that the resilience indicator *r*_auto_ showed moderate genetic variability and favourable genetic correlations with yearling weight, reproductive traits and pre‐weaning survival. This indicates its potential for inclusion in animal breeding programmes for the Nellore breed, aiming at the selection of heifers with a higher recovery rate after being exposed to environmental challenges in extensive rearing environments. The LnVar also seems to be an interesting indicator for selecting resilient heifers, as it showed the highest heritability estimates and the strongest favourable genetic correlations with HP However, this indicator showed unfavourable genetic correlations with YW and WBS, requiring further studies to understand the potential relationship between this indicator and other weight‐related traits in beef cattle. Conversely, the skewness indicator presented low heritability estimates, suggesting a low genetic gain through selection. Additionally, it showed unfavourable genetic correlations with yearling weight and low genetic correlation estimates with reproductive traits, which seem to limit its inclusion as a resilience indicator in animal breeding programmes.

## 4 Conclusions

The resilience indicators LnVar and *r*_auto_ exhibited moderate to high heritability estimates, supporting their potential inclusion in breeding programmes aimed at enhancing the resilience of heifers to environmental challenges in extensive production systems for Nellore cattle. Favourable genetic correlations between *r*_auto_ and yearling weight, heifer pregnancy, calf performance and pre‐weaning survival suggest that selecting heifers with faster recovery rates after disturbances may enhance both productivity and fertility. In contrast, the skewness indicator showed low heritability estimates and unfavourable or null genetic correlations with economically important traits, indicating limited potential for use in breeding programmes for Nellore cattle. Overall, the results highlight a positive association between resilience during the growth phase and reproductive success, suggesting that more resilient heifers are more likely to get pregnant during their first breeding season and to conceive offspring with a higher probability of surviving until weaning. Future research should focus on validating these indicators across diverse environmental conditions and genetic backgrounds to support broader implementation in tropical beef cattle populations.

## Conflicts of Interest

The authors declare no conflicts of interest.

## Supporting information


Tables S1–S4.

## Data Availability

The data can be requested by contacting JNSGC (jgcyrillo@sp.gov.br) and MEZM (mezmercadante@gmail.com) upon a reasonable request for research purposes and with permission of the experimental breeding programme (Nelore‐IZ).
