# Supplementary material for: Genetic Parameters of Resilience Indicators Across Growth in Beef Heifers and Their Associations With Weight, Reproduction, Calf Performance and Pre‐Weaning Survival
Source: J Anim Breed Genet. 2025 Jun 23;143(1):79–91. doi: 10.1111/jbg.70001 (PMC12686759; doi:10.1111/jbg.70001)
Supplement: Supplementary file 1 — Tables S1–S4. [file JBG-143-79-s001.docx]

**Table S1.** Number of animals and single-nucleotide polymorphism (SNP) markers for each panel used for genotyping Nellore cattle.

|  | | | | | |  |
| --- | --- | --- | --- | --- | --- | --- |
| **Panel** | **Males** | **Females** | **Total of animals** | | **No. SNP** | |
| Illumina BovineHD BeadChip  (San Diego, CA, USA) | 468 | 312 | 780 | 777,962 | | |
| GeneSeek® Profiler 75 K - Indicine (Lincoln, NE, USA) | 790 | 528 | 1,318 | 74,153 | | |
| GGP Indicus 50 K  (Lincoln, NE, USA) | 483 | 645 | 1,128 | 54,791 | | |

**Table S2.** Comparison of growth models to describe the weight of Nellore heifers from birth to yearling.

| **Model** | **AIC** | **BIC** | **LogL** |
| --- | --- | --- | --- |
| Repeated measures | 316,230 | 316,297 | -158,107 |
| Brody | 325,601 | 325,635 | -162,796 |
| Legendre 2 | 311,104 | 311,155 | -155,543 |
| Legendre 3 | 301,051 | 301,110 | -150,518 |
| Legendre 4 | 300,206 | 300,273 | -150,095 |
| Legendre 5 | 294,319 | 294,395 | -147,150 |
| Legendre 6* | 293,038 | 293,122 | -146,509 |

Linear model with repeated measures, Brody non-linear growth model, Legendre orthogonal polynomials of variable orders. *Best-fitting model based on evaluation criteria: AIC (Akaike Information Criterion), BIC (Bayesian Information Criterion), and LogL (log-likelihood).

**Table S3.** Comparison of growth models to describe the weight of Nellore heifers from birth to first breeding.

| **Model** | **AIC** | **BIC** | **LogL** |
| --- | --- | --- | --- |
| Repeated measures | 361,109 | 361,143 | -180,550 |
| Brody | 353,366 | 353,434 | -176,675 |
| Legendre 2 | 349,834 | 349,885 | -174,911 |
| Legendre 3 | 347,464 | 347,523 | -173,725 |
| Legendre 4 | 344,226 | 344,294 | -172,105 |
| Legendre 5 | 334,193 | 334,269 | -167,087 |
| Legendre 6* | 332,653 | 332,738 | -166,316 |

Linear model with repeated measures, Brody non-linear growth model, Legendre orthogonal polynomials of variable orders. *Best-fitting model based on evaluation criteria: AIC (Akaike Information Criterion), BIC (Bayesian Information Criterion), and LogL (log-likelihood).

**Table S4.** Comparison of growth models to describe the weight of Nellore heifers from birth to weaning of the first calf.

| **Model** | **AIC** | **BIC** | **-LogL** |
| --- | --- | --- | --- |
| Repeated measures | 420,118 | 420,153 | -210,055 |
| Brody | 418,829 | 418,898 | -209.406 |
| Legendre 2 | 402,013 | 402,065 | -201,000 |
| Legendre 3 | 401,405 | 401,465 | -200,695 |
| Legendre 4 | 396,539 | 396,608 | -198,261 |
| Legendre 5 | 395,422 | 395,500 | -197,702 |
| Legendre 6* | 395,418 | 395,504 | -197,699 |

Linear model with repeated measures, Brody non-linear growth model, Legendre orthogonal polynomials of variable orders. *Best-fitting model based on evaluation criteria: AIC (Akaike Information Criterion), BIC (Bayesian Information Criterion), and LogL (log-likelihood).
